# Magnetic Resonance Imaging Findings in High School Football Players: Brain and Cervical Spine

**DOI:** 10.1089/neur.2021.0026

**Published:** 2022-03-15

**Authors:** Hon J. Yu, Lara Wadi, Irene Say, Annlia Paganini-Hill, Daniel Chow, Arash Hosseini Jafari, Saifal-Deen Farhan, Shane Rayos Del Sol, Osama Mobayed, Andrew Alvarez, Anton Hasso, Scott Shunshan Li, Hung Do, Dawn Berkeley, Yu-Po Lee, Lydia Min-Ying Su, Charles Rosen, Mark Fisher

**Affiliations:** ^1^ Department of Radiological Sciences, University of California Irvine, Irvine, California, USA.; ^2^ Department of Neurology, University of California Irvine, Irvine, California, USA.; ^3^ Department of Orthopedic Surgery, University of California Irvine, Irvine, California, USA.; ^4^ Canon Medical Systems USA, Tustin, California, USA.; ^5^ Department of Pathology & Laboratory Medicine, University of California Irvine, Irvine, California, USA.

**Keywords:** adolescent, concussion, disc degeneration, football, MRI, spinal cord injury, sport-related injury, traumatic brain injury

## Abstract

Football exposes its players to traumatic brain, neck, and spinal injury. It is unknown whether the adolescent football player develops imaging abnormalities of the brain and spine that are detectable on magnetic resonance imaging (MRI). The objective of this observational study was to identify potential MRI signatures of early brain and cervical spine (c-spine) injury in high school football players. Eighteen football players (mean age, 17.0 ± 1.5 years; mean career length, 6.3 ± 4.0 years) had a baseline brain MRI, and 7 had a follow-up scan 9–42 months later. C-spine MRIs were performed on 11 of the 18 subjects, and 5 had a follow-up scan. C-spine MRIs from 12 age-matched hospital controls were also retrospectively retrieved. Brain MRIs were reviewed by a neuroradiologist, and no cerebral microbleeds were detected. Three readers (a neuroradiologist, a neurosurgeon, and an orthopedic spine surgeon) studied the cervical intervertebral discs at six different cervical levels and graded degeneration using an established five-grade scoring system. We observed no statistically significant difference in disc degeneration or any trend toward increased disc degeneration in the c-spine of football players as compared with age-matched controls. Further research is needed to validate our findings and better understand the true impact of contact sports on young athletes.

## Introduction

Football is a collision sport that exposes its players to traumatic brain and spinal injury. Between 2010 and 2016, >50,000 emergency department visits for persons under 18 years of age for non-fatal traumatic brain injury (TBI) were related to playing football, more than any other sport according to the Centers for Disease Control and Prevention.^1^ An estimated 10–15% of professional football players sustain c-spine injuries, ranging from cervical nerve root injury to spinal cord injury.^2,3^ Moreover, in high school athletics, the rate of c-spine injury is highest in football (10.10 per 100,000 athletic exposures) compared with an all-sport average of 3.04 per 100,000 exposures.^4^ Although there has been significant research in traumatic c-spine injury in professional football players,^5^ our understanding of the health of the c-spine in high school football players is limited. Specifically, it is unknown whether the adolescent football player develops any detectable imaging abnormalities on magnetic resonance imaging (MRI) of the c-spine. Previous research has identified c-spine x-ray abnormalities, including disc space narrowing, osteophyte formation, and vertebral body fractures, in football players at a rate ranging from 3.2% in high school sophomores to 32% in college freshmen.^6^

In professional National Football League players, repetitive direct TBI has been linked to chronic traumatic encephalopathy, a neurodegenerative disease characterized by marked neuropsychological decline in executive function, mood, and cognition.^7^ However, risks of cumulative concussive and subconcussive TBI to adolescent football athletes are not only unknown, but unmeasured. To our knowledge, the developing brain of an adolescent football player has not been profiled for early signatures of TBI on MRI.

Our observational study was designed to study high school football players for MRI signatures of early injury to the brain and c-spine using readily available clinical imaging protocols. Identification of detectable structural changes on MRI in this group may further our understanding of the effects of repetitive TBI and neck trauma on the developing brain and c-spine. Further, recognition of such MRI signatures may influence return-to-play guidelines, the role for neuroimaging screening, and early intervention to prevent permanent, potentially devastating neurological dysfunction for the young athlete.

## Methods

### Subjects

This study was approved by the University of California Irvine Institutional Review Board, and informed consent was obtained from all participants. The study subjects were male students from a local high school who had played at least one season of high school football. Exclusion criteria were the following: age younger than 13 years or older than 22 years and contraindications for MRI such as severe claustrophobia. Subjects reported their age, length of football career, player position, and history of concussion.

Eighteen players received an initial brain MRI; 7 of these had a follow-up (F/U) brain MRI. Eleven of the 18 players also received an initial c-spine MRI; 5 received a F/U c-spine MRI. The time interval between the initial and F/U MRIs ranged from 9 to 42 months. After IRB approval, the c-spine MRIs performed between 2015 and 2020 of 12 patients 14–19 years were retrieved retrospectively from our hospital database for use as age-matched controls. Controls were male patients with multiple sclerosis (MS) with no documented history of TBI or neck trauma, undergoing surveillance MRI. After excluding 1 player's F/U c-spine MRI because of severe motion artifact, a total of 25 brain and 15 c-spine MRI studies from players and 12 c-spine MRI studies from controls were evaluated.

### Magnetic resonance imaging

MRI studies were performed on a 3 Tesla scanner (Vantage Galan; Canon Medical Systems, Otawara, Japan) using a 32-channel dedicated receive-only head coil for the brain, and a 16-channel receive-only head/neck coil for the c-spine. The brain MRI protocol included a three-dimensional (3D)/T1-weighted (T1w) image using a magnetization-prepared rapid gradient echo sequence (repetition time [TR]/echo time [TE]/inversion time [TI] = 2300/3.2/900 ms; voxel-size = 1 mm^3^), a 3D-flow-sensitive black blood (FSBB) sequence (TR/TE = 29.2/20 ms; voxel size = 0.5 × 0.5 × 1 mm^3^), which has similar contrast to susceptibility-weighted imaging (SWI), a 3D-T2w (T2-weighted) image using single-shot fast advanced spin echo (FASE; TR/TE = 3000/352 ms; voxel size = 1 mm^3^), and a 3D-T2w-FLAIR (fluid-attenuated inversion recovery) using a FASE sequence (TR/TE/TI = 6000/352/2000 ms; voxel size = 1 mm^3^). The C-spine MRI protocol included fast spin-echo–based sagittal T2w scans with (TR/TE = 3200/60 ms; voxel-size = 0.82 × 0.65 × 3 mm^3^) and without fat suppression (TR/TE = 3000/90 ms; voxel size = 0.82 × 0.65 × 3 mm^3^) and a gradient-echo–based multi-echo T2*w scan in axial orientation (TR/mean TE = 725/11.5 ms; voxel size = 0.78 × 0.78 × 3 mm^3^). The overall duration of both the brain and c-spine MRI was ∼1 h.

### Clinical image assessment of players

Brain and c-spine images were evaluated for identification of any abnormality and for anatomical conformity compared with those from healthy, age-matched persons. They were evaluated on a clinical Picture Archiving and Communications System by a team consisting of an experienced neuroradiologist (for brain and c-spine MRIs) and fellowship-trained orthopedic and neurosurgical spine surgeons (for c-spine MRIs). Brain images were examined specifically for the presence of cerebral microbleeds (CMB) using the FSBB sequence, which is particularly sensitive to the detection of blood products. We also looked for other abnormalities such as acute infarct, gliosis, hemorrhage, mass lesions, and hydrocephalus. Cervical images were examined for swelling in prevertebral soft tissue, evidence of traumatic disc injury, and abnormal appearance in vertebral bodies, cervical cord, and paraspinal soft tissues.

### Assessment of cervical intervertebral disc degeneration

**FIG. 1.**
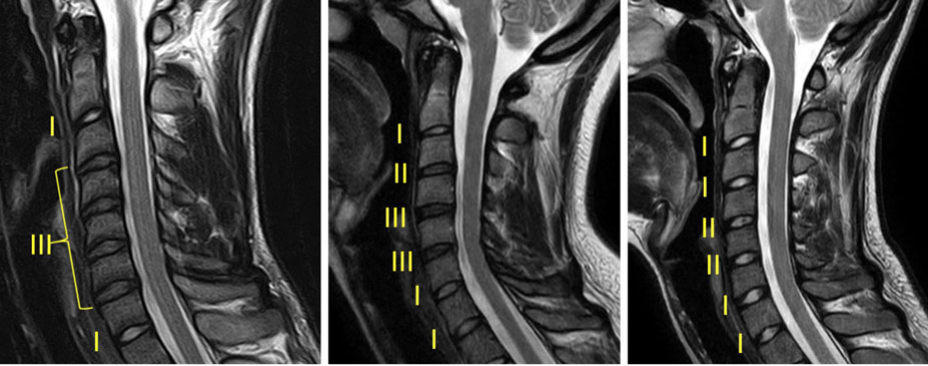
Sample c-spine images demonstrating various disc degeneration grades (from left to right: player and 2 controls).

Deidentified and randomized cervical images were transferred in the Digital Imaging and Communications in Medicine format to a personal computer, and the degenerative grading of the cervical intervertebral discs (IVD) was performed offline. The degenerative grade of each cervical level (C2/3, C3/4, C4/5, C5/6, C6/7, and C7/T1) was assessed by three readers (a neuroradiologist, a neurosurgeon, and an orthopedic spine surgeon) using ImageJ software^8^ on the mid-sagittal slice of T2w images based on a five-level grading system (Table 1), as described in previous literature.^9^ The mid-sagittal slice was selected by visualization of the atlas (C1), and it was ensured that the same sagittal T2w image was used by all three readers for degenerative grading in a given c-spine MRI. The three readers had no interaction for consensus or training before or during their reading for this study. Sample images of a player and 2 controls depicting various degenerative grades are shown in Figure 1.

**Table 1.** Player Characteristics and Numbers Participating in Different Imaging Studies

	All players	Self-reported history of concussion	
		Yes	No
Age at initial MRI (years) (mean ± standard deviation)	17.0 ± 1.5	17.0 ± 1.3	17.0 ± 1.7
Career length (years) (mean ± standard deviation)	6.3 ± 4.0	7.1 ± 4.4	5.4 ± 3.5
Brain MRI			
Initial study	18	9	9
Follow-up study	7	4	3
C-spine MRI			
Initial study	11	5	6
Follow-up study	5	3	2

MRI, magnetic resonance imaging.

### Statistical analysis

Means and standard deviations (SD) of continuous variables and proportions of categorical variables were calculated. The interobserver agreement of the degenerative grading of cervical IVD was estimated using kappa statistics. The final degenerative IVD grade at each of the six cervical levels in each subject was determined based on the mode (most frequent) grade of the three readers. In case of three different grades, the middle grade was selected as the final grade. Grade distribution at each cervical level was compared between player and control groups using box plots generated with SPSS statistical software (version 25.0; IBM SPSS, IBM Corporation, Armonk, NY). The Mann-Whitney U test was performed to compare the median IVD grade between player and control groups as well as between players with and without a concussion history. A *p* value <0.05 was considered to be significant. All statistical analyses were carried out using Matlab (version 9.7; The MathWorks, Inc., Natick, MA).

## Results

### Subject demographics

The 18 football players ranged in age from 15 to 20 years (including repeat scans done after high school) with a mean ± SD 17.0 ± 1.5 and career length from 2 to 13 years (6.3 ± 4.0) at the time of their initial MRI (Table 2). Player positions included running back (*n* = 5), lineman (*n* = 5), safety (*n* = 2), cornerback (*n* = 2), defensive end (*n* = 1), wide receiver (*n* = 1), kick returner (*n* = 1), and unknown (*n* = 1). Mean career length (±SD) of subjects with a concussion history (7.1 ± 4.4 years) was longer than that of subjects without a concussion history (5.4 ± 3.5 years), but the difference was not statistically significant. Controls ranged in age from 14 to 19 years, and their mean age (17.7 ± 1.5 years) did not differ from that of players (17.1 ± 1.8). Controls included 12 male MS patients undergoing surveillance MRI without any known history of TBI or neck trauma.

**Table 2.** Grading System for Cervical Intervertebral Disc Degeneration

Grade	Nucleus signal intensity	Nucleus structure	Distinction of nucleus and annulus	Disc height
I	Hyperintense	Homogeneous, white	Clear	Normal
II	Hyperintense	Inhomogeneous w/ horizontal band, white	Clear	Normal
III	Intermediate	Inhomogeneous, gray to black	Unclear	Normal to decreased
IV	Hypointense	Inhomogeneous, gray to black	Lost	Normal to decreased
V	Hypointense	Inhomogeneous, gray to black	Lost	Collapsed

### Clinical image assessment of players

**FIG. 2.**
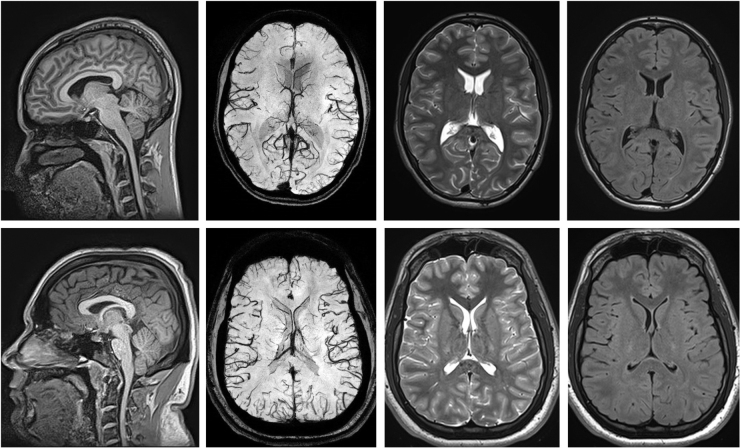
Sample brain images from a player without (top row) and another player with (bottom row) concussion history. The images from left to right are 3D-T1w, 3D-FSBB, 3D-T2w, and 3D-FLAIR. All images are read as normal. 3D, three-dimensional; FLAIR, fluid-attenuated inversion recovery; FSBB, flow sensitive black blood; T1w, T1-weighted; T2w, T2-weighted.

**FIG. 3.**
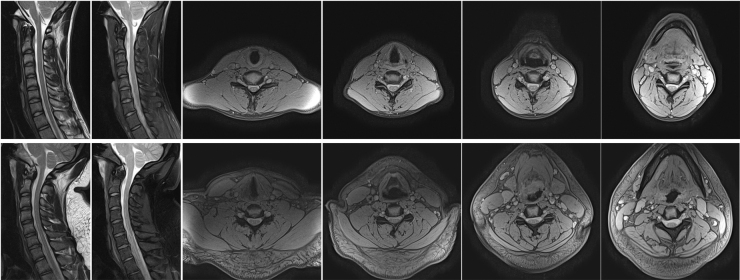
Sample c-spine images acquired using various sequences from a player without (top row) and with (bottom row) concussion history. The presented images from left to right are: T2w in sagittal orientation; fat suppressed T2w in sagittal orientation; and multi-echo T2*w in axial orientation at four different locations. The assigned grades in the player without concussion history were II, III, II, III, II, and I at C2/3, C3/4, C4/5, C5/6, C6/7, and C7/T1, respectively; the assigned grades in the player with concussion history were I, III, III, III, III, and I at C2/3, C3/4, C4/5, C5/6, C6/7, and C7/T1, respectively. T2w, T2-weighted.

No evidence of TBI or cerebral abnormalities, including contusion, subarachnoid, subdural, epidural, or petechial hemorrhages, edema, or skull fracture, was observed in any player's brain MRI. We did not detect any CMB on analysis of players' MRI-FSBB sequences. Sample brain images from 2 players, 1 with and 1 without a concussion history, are shown in Figure 2. None of the subjects' c-spine MRI demonstrated abnormalities typically associated with acute or traumatic spine injury, such as soft-tissue swelling, vertebral compression fracture, and myelomalacia. However, a number of the analyzed cervical IVD showed substantial degeneration (see next paragraph). Sample c-spine images from the same 2 players whose brain images are shown in Figure 2 are shown in Figure 3.

### Cervical intervertebral disc degeneration

**FIG. 4.**
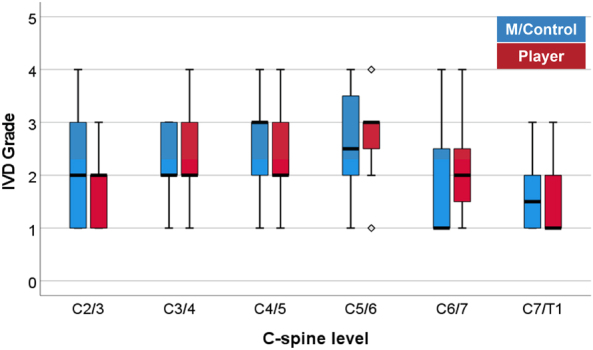
Box plot of disc degeneration grades comparing players and controls at each cervical level. On each box, the thick horizontal mark indicates the median (the 50th percentile), and the bottom and top edges of the box represent the 25th and 75th percentiles, respectively. Whiskers extend to the most extreme data points not considered outliers, and the outliers are plotted individually using the 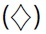 symbol.

A total of 138 cervical discs from 23 c-spine data sets were graded by each of the three readers for comparison between controls and players. They consisted of 66 discs from the 11 c-spine MRIs belonging to players (using only initial scans and excluding four F/U c-spine MRIs) and 72 discs from the 12 controls' data sets. Interobserver agreement among readers ranged from fair to moderate (kappa = 0.34–0.51). Table 3 gives the number and percentage of players and controls assigned to grade I–V at each of the six cervical levels using the final degenerative IVD grade. The total number of discs in each of the five grades was averaged over the six cervical levels, and the percentages assigned to individual grades for players and controls are listed in Table 4. Distribution in disc degeneration grades between the player and control groups at each cervical level was also compared using box plots (Fig. 4). There was no difference in median grade observed between players and controls at any cervical level. Likewise, no difference in median grade was observed between players with and without a concussion history at any cervical level. Also, there was no statistically significant correlation between mean career length and disc grade at any cervical level (*p* > 0.36).

**Table 3.** Number (Percentage) of Players (*P*) and Controls (C) with Grades I–V at Each of the Six Cervical Levels: C2/3, C3/4, C4/5, C5/6, C6/7, and C7/T1

	C2/3		C3/4		C4/5		C5/6		C6/7		C7/T1	
	*P*	C	*P*	C	*P*	C	*P*	C	*P*	C	*P*	C
Grade I	5 (45%)	4 (33%)	1 (9%)	1 (8%)	1 (9%)	2 (17%)	2 (18%)	2 (17%)	3 (27%)	7 (58%)	7 (64%)	6 (50%)
Grade II	5 (45%)	4 (33%)	5 (45%)	6 (50%)	6 (5%)	2 (17%)	1 (9%)	4 (33%)	5 (45%)	2 (17%)	2 (18%)	4 (33%)
Grade III	1 (9%)	3 (25%)	4 (36%)	5 (42%)	3 (27%)	6 (50%)	6 (55%)	3 (25%)	2 (18%)	2 (17%)	2 (18%)	2 (17%)
Grade IV	0	1 (8%)	1 (9%)	0	1 (9%)	2 (17%)	2 (18%)	3 (25%)	1 (9%)	1 (8%)	0	0
Grade V	0	0	0	0	0	0	0	0	0	0	0	0

**Table 4.** Percentage of Total Cervical Discs Assigned to Each of the Five Grades in Players and Controls

	Grade I	Grade II	Grade III	Grade IV	Grade V
Players	29	36	27	8	0
Controls	22	22	21	7	0

**FIG. 5.**
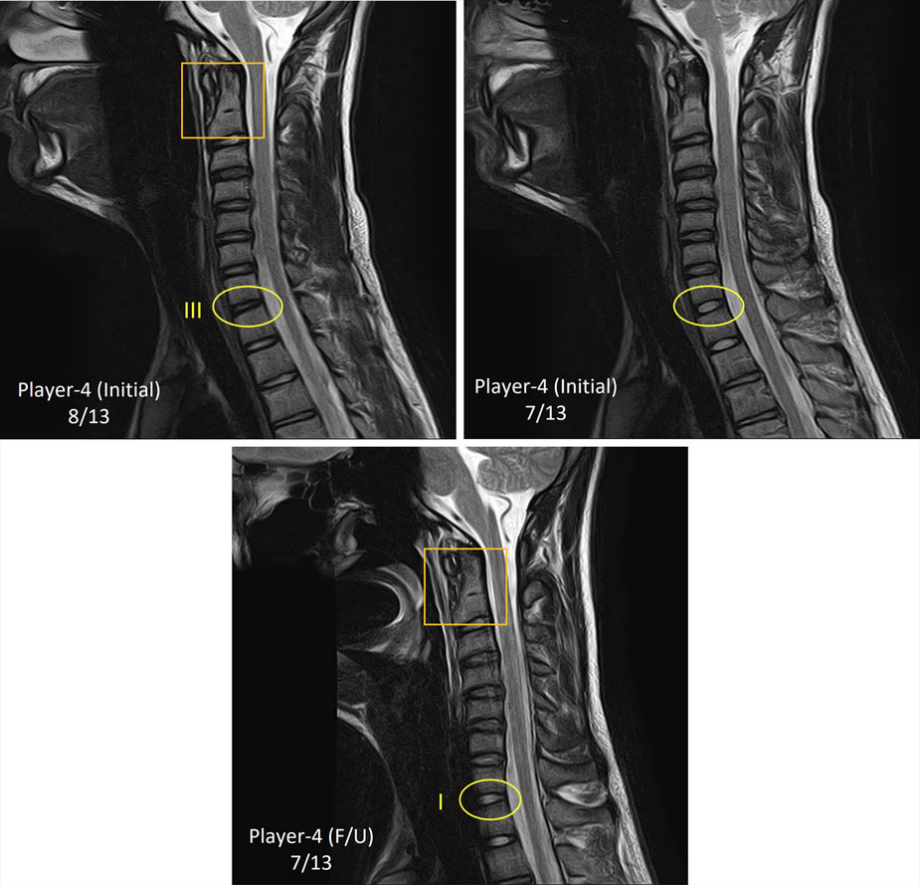
Mid-sagittal T2w image (top left) read by the readers and the image from the adjacent slice (top right) in the initial c-spine MRI and the image in the follow-up c-spine MRI (bottom) of one of the four players with repeated c-spine MRI. Selection of the sagittal slice for degenerative reading is based on visualization of the atlas (C1, orange square). In the initial scan of this subject, the adjacent slice would have been the true mid-sagittal for the cervical disc at the C7/T1 level and given a lower degenerative grade (very likely grade I) than the assigned (grade III). In the follow-up c-spine MRI, the mid-sagittal slice that was selected for the read (orange square) is the true mid-sagittal slice for the cervical disc at the C7/T1 level and yielded grade I.

Initial and F/U disc degeneration grades of 4 players who had repeated c-spine MRI were separately compared (Table 5). Of the total of 24 cervical discs, 20 discs (83%) had grade I or II and four discs (17%) had grade III or IV assigned at the initial scan. Eight of the 24 discs (33%) showed an increase of one grade in the F/U MRI in comparison with the initial MRI. Eleven discs (46%) showed no change in grade, and three discs (13%) showed a decrease of one grade. A grade change of more than one from initial to F/U MRI was noted at a single cervical level in each of 2 players: Player 3 (from grade I to III at C3/4) and player 4 (from grade III to I at C7/T1). However, closer examination revealed that a less-than-consistent selection of mid-sagittal slice for the disc at a given cervical level between initial and F/U scans was likely responsible, that is, a disc sampling discrepancy. Unless the disc is highly degenerated (grade IV or V), the sagittal slice cutting through the middle of the nucleus pulposus, that is, the true mid-sagittal slice, would likely give a lower grade than the adjacent slice that contains more of the anulus fibrosus, which appears darker in T2w than nucleus pulposus. This sampling discrepancy and resulting difference in disc grade are demonstrated visually in Figure 5.

**Table 5.** Degenerative Grades of Cervical Discs on Initial and F/U Scans of 4 Players Who Had Repeated Cervical MRIs

Subject	Time point	C2/3	C3/4	C4/5	C5/6	C6/7	C7/T1
Player 1	Initial	I	II	I	I	I	I
	F/U	II	III	I	I	II	I
Player 2	Initial	I	II	II	I	I	I
	F/U	II	I	II	I	I	I
Player 3	Initial	II	I	III	IV	II	II
	F/U	III	III	III	III	II	I
Player 4	Initial	II	III	II	II	II	III
	F/U	II	III	III	III	III	I

F/U, follow-up; MRI, magnetic resonance imaging.

## Discussion

In this observational study, we surveyed high school football players with and without a history of concussion, with different playing positions and varying years of experience for possible MRI signatures of early injury to the brain and c-spine. We did not detect CMB on the brain MRIs of football players, regardless of concussion history. Likewise, there was no statistically significant difference in disc degeneration or any trend toward increased disc degeneration in the c-spine of football players as compared with age-matched controls.

Little data on the early neuroimaging findings in young athletes exist. Though it is well established that TBI can lead to the development of MRI-detectable abnormalities, such as CMB,^10,11^ it is unknown how direct repetitive TBI affects the development of the adolescent brain and c-spine. CMB are exceedingly rare in young people without a history of trauma and have long served as a surrogate marker for TBI. They are associated with the accumulation of blood products, such as hemosiderin, which are best detected by T2*-weighted gradient echo or SWI-MRI sequences as small hypointense lesions.^10,13^ Whereas clinical outcomes associated with traumatic CMB may vary,^10,15^ reduction in CMB volume over time has been associated with improved clinical outcomes.^16^ However, the long-term effects of persistent CMB secondary to TBI are poorly understood.

In this study, our results demonstrated the absence of CMB and other detectable abnormalities typically associated with sports-related injuries in the brain of football players, regardless of concussion history. Our negative findings are consistent with previously published studies of sports-related injury to the brain of children and young adults. In a pediatric sample (ages 9–15 years) with mild TBI in a level 1 trauma center of a regional children's hospital, presence of CMB was detected by SWI only in children with falls or TBI associated with a motor vehicle accident, and not in those with sports-related injuries.^17^ In another study of 151 cases of pediatric sports-related concussion, CMB were detected in only 1 of 36 persons who underwent MRI.^18^ Jarrett and colleagues examined 40 collegiate ice-hockey players (mean age, 21.2 ± 3.1 years) over a season, and no CMB was detected on SWI.^19^ However, the presence or absence of CMB may not completely describe TBI. For example, Adler and colleagues found that 11 former Division 1 football players had statistically significant lower cortical thickness in both the frontal and temporal cortex compared with demographically similar track-and-field athletes.^20^

Findings from such studies may reassure young athletes and their families that current return-to-play protocols and protective head gear are effective. Conversely, this may frustrate young symptomatic athletes recovering from post-concussive TBI given that these studies uncover marked limitations in our current clinical MRI protocols for sports-related concussion.

Many studies have suggested that young athletes participating in sports with moderate or severe strain on the spine run a high risk of developing disc degeneration and other abnormalities in the thoracolumbar spine observable on MRI compared with controls.^21–24^ Additionally, incidence of such degenerative changes has been reported to be higher during growth spurts.^22,23,25,26^ C-spine injury rates among high school athletes are the highest in football compared to other sports,^4^ and MRI is well suited to imaging of the c-spine for the evaluation of sport-related injuries.^27^ However, few reports investigating such degenerative changes in discs of the c-spine in young athletes using MRI have been published. More than 20 years ago, Berge and colleagues^28^ reported increased degeneration in 56% of cervical discs in rugby players ≥20 years of age compared with 15% in age-matched controls. The same study noted no degenerated discs in younger rugby players (16–19 years of age) or in their age-matched controls. Their arbitrary definition of disc degeneration as a hyposignal on T2w scans, based on poor image quality from using an inferior radiofrequency coil, makes additional inferences from the study difficult, however.

In our study subjects of football players, no statistically significant difference in disc degeneration or any trend toward increased disc degeneration was observed when compared with age-matched controls. Players demonstrated increased disc degeneration at two cervical levels (median grade III vs. II ½ at C5/6 and median grade II vs. I at C6/7), the same at two cervical levels (median grade II at C2/3 and C3/4), and decreased disc degeneration at two remaining cervical levels (median grade II vs. III at C4/5 and median grade I vs. I ½ at C7/T1). In a 10-year follow-up study of asymptomatic subjects, Okada and colleagues^29^ reported that only age was significantly associated with progression of degeneration of c-spine and there was no significant correlation between any of the degenerative MR findings and other factors, including sex, smoking, alcohol, sports, or body mass index. In their study, the sports group consisted of the subjects who took part in regular recreational sports activities at least once a week during the preceding 10-year period and made up ∼15% of the total number of study subjects (*n* = 223).

More recently, Abdalkader and colleagues investigated the prevalence of spinal disc degenerative changes using MRI and the same five-grade scoring system as our study in athletes participating in the Rio de Janeiro 2016 Summer Olympics games.^30^ Using 21 MRI studies from 5 female and 16 male athletes, the researchers noted that cervical degenerative changes were predominantly observed in men and >30 years of age. They also noted that shooters and judo athletes were the most affected by mild degeneration (grade II or III) whereas athletics, boxers, and swimmers were the athletes most affected by moderate degeneration (grade IV). In their youngest age group for the c-spine (between 20 and 30 years of age), disc degeneration ranging from mild to moderate (grade II–IV) was observed in athletes from three sports, including swimming (F/M = 0/5), soccer (F/M = 0/5), and gymnastics (F/M = 1/5). Despite its novel nature of using elite athletes from various disciplines as subjects, the study suffers from a lack of subjects in their teens (for the c-spine) as well as possible sample-size/selection bias (subjects were volunteers who had pain in the neck or mid or lower back) for us to draw any inferences relevant to our findings.

Our study has several limitations. Because of reasons such as scheduling conflict, relocation after graduation, or refusal to participate, some of the players did not have repeat MRI studies. Only 7 of 18 players completed F/U brain MRIs, and 5 of 11 players completed F/U c-spine MRIs, resulting in a small sample size. In addition, only fair-to-moderate interobserver agreement, with kappa scores of 0.34–0.51, was noted in the interpretation of cervical intervertebral disc degeneration in our study. This is lower than modest to substantial kappa values (0.44–0.67) reported in the literature for visual assessment of cervical spine disc degeneration using MRI based on slightly different scoring systems.^31–33^ It is likely that training, in addition to individual raters' review of the disc grade criteria, would have improved interobserver agreement.^34^ The use of MS patients' c-spine MRI for the comparison group is another limitation. Although age and sex matched, patients' background and medical conditions (other than absence of TBI or neck trauma) were unknown and may have had an impact on degenerative grades in our control group. Finally, because of the relatively small size of cervical IVD, the location or selection of the sagittal slice on which visual assessment is made for disc degeneration becomes more critical. Although the c-spine sagittal scan was performed with an angulation in coronal plane to account for any less-than-straight neck orientation of the subject inside the magnet, 2 players with F/U MRI demonstrated that disc grades that differ by two steps are possible for a given cervical IVD simply because of the choice of which of the two adjacent sagittal slices was used for assessment. This should be carefully considered in any longitudinal study that investigates changes in cervical IVDs.

## Conclusion

In this observational study, there were no abnormalities on brain MRIs of high school football players and no statistically significant differences in c-spine MRIs between football players and age-matched male controls based on clinical MRI protocols.

## Abbreviations Used

3D: three-dimensional
CMB: cerebral microbleed
FASE: fast advanced spin echo
FLAIR: fluid-attenuated inversion recovery
FSBB: flow-sensitive black blood
F/U: follow-up
IRB: Institutional Review Board
IVDs: intervertebral discs
MRI: magnetic resonance imaging
MS: multiple sclerosis
NP: nucleus pulposus
SD: standard deviation
SWI: susceptibility-weighted imaging
T1w: T1-weighted
T2w: T2-weighted
TBI: traumatic brain injury
TE: echo time
TI: inversion time
TR: repetition time

## References

[1] Haileyesus, T., and Breiding, M.J. (2019). Emergency department visits for sports- and recreation-related traumatic brain injuries among children—United States, 2010–2016. MMWR Morb. Mortal. Wkly. Rep. 68, 237–242. 10.15585/mmwr.mm6810a2 PMC6421963 30870404

[2] Rihn, J.A., Anderson, D.T., Lamb, K., Deluca, P.F., Bata, A., Marchetto, P.A., Neves, N., and Vaccaro, A.R. (2009). C-spine injuries in American football. Sports Med. 39, 697–708. 19691361 10.2165/11315190-000000000-00000

[3] Thomas, B.E., McCullen, G.M., and Yuan, H.A. (1999). C-spine injuries in football players. J. Am. Acad. Orthop. Surg. 7, 338–347. 10504360 10.5435/00124635-199909000-00006

[4] Meron, A., McMullen, C., Laker, S.R., Currie, D., and Comstock, R.D. (2018). Epidemiology of C-spine injuries in high school athletes over a ten-year period. PM R 10, 365–372. 28919185 10.1016/j.pmrj.2017.09.003

[5] Torg, J.S. (2009). C-spine injuries and the return to football. Sports Health 1, 376–383. 23015896 10.1177/1941738109343161 PMC3445180

[6] Albright, J.P., Moses, J.M., Feldick, H.G., Dolan, K.D., and Burmeister, L.F. (1976). Nonfatal c-spine injuries in interscholastic football. JAMA 236, 1243–1245. 989067

[7] Mez, J., Daneshvar, D.H., Kiernan, P.T., Abdolmohammadi, B., Alvarez, V.E., Huber, B.R., Alosco, M.L., Solomon, T.M., Nowinski, C.J., McHale, L., Cormier, K.A., Kubilus, C.A., Martin, B.M., Murphy, L., Baugh, C.M., Montenigro, P.H., Chaisson, C.E., Tripodis, Y., Kowall, N.W., Weuve, J., McClean, M.D,. Cantu, R.C., Goldstein, L.E., Katz, D.I., Stern, R.A., Stein, T.D., and McKee, A.C. (2017). Clinicopathological evaluation of chronic traumatic encephalopathy in players of American Football. JAMA 318, 360–370. 28742910 10.1001/jama.2017.8334 PMC5807097

[8] National Institute of Health, Bethesda, MD, USA. Image Processing and Analysis in Java (ver 1.51) [Online]. Available: https://imagej.nih.gov/ij/, Accessed on: Apr. 21, 2021.

[9] Miyazaki, M., Hong, S.W., Yoon, S.H., Morishita, Y., and Wang, J.C. (2008). Reliability of a magnetic resonance imaging-based grading system for cervical intervertebral disc degeneration. J. Spinal. Disord. Tech. 21, 288–292. 18525490 10.1097/BSD.0b013e31813c0e59

[10] Liu, J., Xia, S., Hanks, R., Wiseman, N., Peng, C., Zhou, S., Haacke, E.M., and Kou, Z. (2016). Susceptibility weighted imaging and mapping of micro-hemorrhages and major deep veins after traumatic brain injury. J. Neurotrauma 33, 10–21. 25789581 10.1089/neu.2014.3856

[11] Fazekas, F., Kleinert, R., Roob, G., Kleinert, G., Kapeller, P., Schmidt, R., and Hartung, H.P. (1999). Histopathologic analysis of foci of signal loss on gradient-echo T2*-weighted MR images in patients with spontaneous intracerebral hemorrhage: evidence of microangiopathy-related microbleeds. AJNR Am. J. Neuroradiol. 20, 637–642. 10319975 PMC7056037

[12] Liu, A.Y., Maldjian, J.A., Bagley, L.J., Sinson, G.P., and Grossman, R.I. (1999). Traumatic brain injury: diffusion-weighted MR imaging findings. AJNR Am. J. Neuroradiol. 20, 1636–1641. 10543633 PMC7056184

[13] Charidimou, A., Krishnan, A., Werring, D.J., and Rolf Jager, H. (2013). Cerebral microbleeds: a guide to detection and clinical relevance in different disease settings. Neuroradiology 55, 655–674. 23708941 10.1007/s00234-013-1175-4

[14] van der Horn, H.J., de Haan, S., Spikman, J.M., de Groot, J.C., and van der Naalt, J. (2018). Clinical relevance of microhemorrhagic lesions in subacute mild traumatic brain injury. Brain Imaging Behav. 12, 912–916. 28664231 10.1007/s11682-017-9743-6 PMC5990550

[15] Wang, X., Wei, X.E., Li, M.H., Li, W.B., Zhou, Y.J., Zhang, B., and Li, Y.H. (2014). Microbleeds on susceptibility-weighted MRI in depressive and non-depressive patients after mild traumatic brain injury. Neurol. Sci. 35, 1533–1539. 24740482 10.1007/s10072-014-1788-3

[16] Lawrence, T.P., Pretorius, P.M., Ezra, M., Cadoux-Hudson, T., and Voets, N.L. (2017). Early detection of cerebral microbleeds following traumatic brain injury using MRI in the hyper-acute phase. Neurosci. Lett. 655, 143–150. 28663054 10.1016/j.neulet.2017.06.046 PMC5541760

[17] Bigler, E.D., Abildskov, T.J., Goodrich-Hunsaker, N.J., Black, G., Christensen, Z.P., Huff, T., Wood, D.M.G., Hesselink, J.R., Wilde, E.A., and Max, J.E. (2016). Structural neuroimaging findings in mild traumatic brain injury. Sports Med. Arthrosc. Rev. 24, e42–e52. 27482782 10.1097/JSA.0000000000000119 PMC6581215

[18] Ellis, M.J., Leiter, J., Hall, T., McDonald, P.J., Sawyer, S., Silver, N., Bunge, M., and Essig, M. (2015). Neuroimaging findings in pediatric sports-related concussion. J. Neurosurg. Pediatr. 16, 241–247. 26031620 10.3171/2015.1.PEDS14510

[19] Jarrett, M., Tam, R., Hernandez-Torres, E., Martin, N., Perera, W., Zhao, Y., Shahinfard, E., Dadachanji, S., Taunton, J., Li, D.K.B., and Rauscher, A. (2016). A prospective pilot investigation of brain volume, white matter hyperintensities, and hemorrhagic lesions after mild traumatic brain injury. Front. Neurol. 7, 11. 26903944 10.3389/fneur.2016.00011 PMC4751255

[20] Adler, C.M., DelBello, M.P., Weber, W., Williams, M., Duran, L.R.P., Fleck, D., Boespflug, E., Eliassen, J., Strakowski, S.M., and Divine, J. (2018). MRI evidence of neuropathic changes in former college football players. Clin. J. Sport Med. 28, 100–105. 27755011 10.1097/JSM.0000000000000391

[21] Goldstein, J.D., Berger, P.E., Windler, G.E., and Jackson, D.W. (1991). Spine injuries in gymnasts and swimmers. An epidemiologic investigation. Am. J. Sports Med. 19, 463–468. 1962710 10.1177/036354659101900507

[22] Barranto, A., Hellstrom, M., Nyman, R., Lundin, O., and Sward, L. (2006). Back pain and degenerative abnormalities in the spine of young elite divers. A 5-year follow-up magnetic resonance imaging study. Knee Surg. Sports Traumatol. Arthrosc. 14, 907–914. 16416326 10.1007/s00167-005-0032-3

[23] Barranto, A., Hellström, M., Cederlund, C.-G., Nyman, R., and Swärd, L. (2009). Back pain and MRI changes in the thoraco-lumbar spine of top athletes in four different sports: a 15-year follow-up study. Knee Surg. Sports Traumatol. Arthrosc. 17, 1125–1134. 19305975 10.1007/s00167-009-0767-3

[24] Witwit, W.A., Kovac, P., Sward, A., Agnvall, C., Todd, C., Thoreson, O., Hebelka, H., and Barranto, A. (2018). Disc degeneration on MRI is more prevalent in young elite skiers compared to controls. Knee Surg. Sports Traumatol. Arthrosc. 26, 325–332. 28409199 10.1007/s00167-017-4545-3 PMC5754419

[25] Tertti, M., Paajanen, H., Kujala, U.M., Alanen, A., Salmi, T.T., and Kormano, M. (1990). Disc degeneration in young gymnasts. A magnetic resonance imaging study. Am. J. Sports Med. 18, 206–208. 2140492 10.1177/036354659001800216

[26] Rosendahl, K., and Strouse, P.J. (2016). Sports injury of the pediatric musculoskeletal system. Radiol. Med. 121, 431–441. 26838592 10.1007/s11547-015-0615-0

[27] Mintz, D.N. (2004). Magnetic Resonance Imaging of Sports Injuries to the C-spine. Semin. Musculoskelet. Radiol. 8, 99–110. 15085480 10.1055/s-2004-823017

[28] Berge, J., Marque, B., Vital, J.-M., Senegas, J., and Caille, J.-M. (1999). Age-related Changes in the C-spines of front-line rugby players. Am. J. Sports Med. 27, 422–429. 10424210 10.1177/03635465990270040401

[29] Okada, E., Matsumoto, M., Ichihara, D., Chiba, K., Toyama, Y., Fujiwara, H., Momoshima, S., Nishiwaki, Y., Hashimoto, T., Ogawa, J., Watanabe, M., and Takahata, T. (2009). Aging of the C-spine in healthy volunteers. Spine 34, 706–712. 19333104 10.1097/BRS.0b013e31819c2003

[30] Abdalkader, M., Guermazi, A., Engebretsen, L., Roemer, F.W., Jarraya, M., Hayashi, D., Crema, M.D., and Mian, A.Z. (2020). MRI-detected spinal disc degenerative changes in athletes participating in the Rio de Janeiro 2016 Summer Olympics games. BMC Musculoskelet. Disord. 21, 45. 31959161 10.1186/s12891-020-3057-3 PMC6972034

[31] Matsumoto, M., Fujimura, Y., Suzuki, N., Nishi, Y., Nakamura, M., Yabe, Y., and Shiga, H. (1998). MRI of cervical intervertebral discs in asymptomatic subjects. J. Bone. Joint. Surg. 80, 19–24. 10.1302/0301-620x.80b1.7929 9460946

[32] Siivola, S.M., Levoska, S., Tervonen, O., Ilkko, E., Vanharanta, H., and Keinänen-Kiukaanniemi, S. (2002). MRI changes of c-spine in asymptomaticc and symptomatic young adults. Eur. Spine. J. 11, 358–363. 12193998 10.1007/s00586-001-0370-x PMC3610480

[33] Kolstad, F., Myhr, G., Kvistad, K.A., Nygaard, Ø.P., and Leivseth, G. (2005). Degeneration and height of cervical discs classified from MRI compared with precise height measurements from radiographs. Eur. J. Radiol. 55, 415–420. 16129250 10.1016/j.ejrad.2005.02.005

[34] Symmons, D.P., Van Hmert, A.M., Vandenbroucke, J.P., and Valkenburg, H.A. (1991). A longitudinal study of back pain and radiological changes in the lumbar spines of middle aged women. II. Radiolographic findings. Ann. Rheum. Dis. 50, 162–166. 10.1136/ard.50.3.162 PMC1004366 1826598

